# The power of researcher-practitioner partnerships in implementation science: a community case study in sexual violence prevention program evaluation

**DOI:** 10.3389/frhs.2025.1700115

**Published:** 2026-01-12

**Authors:** Caitlin K. Barthelmes, Amanda Childress, Dawn E. Gillis, Chaehyun Lee, Jane G. Stapleton

**Affiliations:** 1 Student Wellness Center, Health and Wellness Division, Dartmouth College, Hanover, NH, United States; 2 Prevention Innovations Research Center, University of New Hampshire, Durham, NH, United States

**Keywords:** program evaluation, higher education, implementation science, prevention, researcher-practitioner partnership, sexual violence

## Abstract

Many institutions of higher education recognize the importance of sexual violence prevention efforts. However, often practitioners tasked with offering prevention efforts lack the time and expertise to assess programs. Researcher-practitioner partnerships offer a solution that provides mutual benefits and is reinforced by implementation science. Through our own experiences in a six-year collaboration between a university research center and a small private college's sexual violence prevention team we discovered the value of researcher-practitioner partnerships. Our partnership focused on evaluating a multi-year, required sexual violence prevention curriculum, currently implementing best-practice skill-building and prevention-techniques for college students at a small private college. Robust evaluation efforts, made possible through the researcher-practitioner partnership, have been essential in gaining key insights and making data-informed improvements to ensure effectiveness of the curriculum. The manuscript provides background on the importance of sexual violence prevention on college campuses and how implementation science and effective researcher-practitioner partnerships can address challenges. Findings from the evaluation project will be shared in future publications, allowing this article to focus on best practices, methodology, and lessons learned related to the researcher-practitioner partnership that align with key implementation science constructs. We aim to offer actionable methods and strategies for other researchers, evaluators, and practitioners to strengthen prevention efforts in higher education settings and beyond.

## 1 Introduction

Across public health settings, prevention programs are often implemented under real-world constraints that limit rigorous evaluation. Practitioners responsible for designing, delivering, and refining these programs frequently lack the time, resources, or technical expertise needed to assess impact, iterate with fidelity, and demonstrate accountability to stakeholders. Additionally, practitioners may not have adequate relationships with evaluators who could provide the needed skills. The result is a persistent evidence to practice gap: promising strategies are deployed, but their effectiveness, reach, and sustainability remain insufficiently measured, hampering improvement and scale (1–3). This tension is visible in campus-based sexual violence (SV) prevention, where institutions face federal compliance, limited resources, and urgent expectations from students, survivors, and policymakers to provide engaging universal training that shows instant, measurable change.

And yet, the need to prevent SV in higher education is critical. Findings from a national multi-campus climate survey indicate that approximately 26.4% of female and 6.8% of male undergraduate students reported unwanted sexual contact, including sexual assault, relationship violence, stalking and harassment and only 45% of bystanders who witnessed an assault intervened (4). Studies have indicated that half of transgender and non-binary individuals report experiencing SV (5–9). The consequences of victimization on mental and physical health as well as academic and career outcomes are severe (10–13) and are heightened for marginalized identities (14–21). These findings highlight the need for a comprehensive SV prevention approach rather than the standard one-time prevention messages delivered during college orientation (22, 23). Given the complexities and reach of negative impacts, public health research highlights the importance of addressing multiple levels of the social ecological model (SEM) including individual, relationship, community, and societal factors in SV prevention efforts (24).

Implementation science offers a pragmatic bridge between what works under controlled conditions and what endures in complex organizational, multi-level environments. Models such as the Consolidated Framework for Implementation Research (CFIR) provide guidance to practitioners and researchers to consider constructs that affect implementation and related research across various domains (25, 26). Implementation science helps translate evidence-based strategies into routine practice by supporting continuous quality improvement, fidelity, and sustainability (27, 28). In university environments where missions, authority over resources, and access to staff time and data shape what is feasible, partnership-based implementation work is especially valuable (29).

Researcher–practitioner partnerships have long been recognized as a vehicle for bridging the gap between research and applied settings (30, 31). In higher education, researcher–practitioner partnerships have supported institutional change by aligning research design with campus realities and promoting co-ownership of data and evaluation processes (28, 32, 33). Working collectively with researchers also provides prevention staff with hands-on experience by participating in research design and data collection, which has been shown to increase practitioner uptake of evidence-based practices as well as contribute to their professional growth (30, 31). These collaborations help translate implementation science principles, such as context sensitivity, iterative feedback, stakeholder engagement, and sustainability into feasible campus practices (34, 35). Thus, partnered implementation science addresses political and ethical realities of evaluation in institutions by pairing practitioner insight with research design and measurement expertise to generate feasible, decision-useful evidence.

Evidence from criminal justice, behavioral health, and violence prevention also show that structured collaboration clarifies roles, improves data access and quality, supports mutual learning, and fosters trust necessary for adaptation without drift (30, 31, 35, 36). Taken together, this literature suggests that researcher–practitioner partnerships, grounded in implementation science, can convert evaluation from a compliance activity into an engine of effective prevention and program improvement.

Our case study extends this body of work by illustrating how a researcher–practitioner partnership can operationalize these constructs to evaluate and refine a multi-year sexual-violence prevention curriculum within a higher education setting. This manuscript reports on a six-year partnership between a university research center and a small private college's Sexual Violence Prevention Project (SVPP) with a goal of providing actionable methods that other campuses and public health organizations can adopt.

## 2 SVPP background

SVPP is a required multi-year curriculum designed to equip undergraduate students with the lifelong skills to build safer and more supportive communities, free from violence. With a comprehensive, developmental, and asset-based approach, SVPP strives to drive cultural change. The curriculum strengthens students' skills around four behavioral outcomes: developing positive relationships and sexual behavior; using power responsibly to foster equity and belonging; intervening as active bystanders; accessing resources, if needed, and supporting those impacted by SV. While many prevention programs remain focused on shifting knowledge and attitudes (27) the SVPP aims to reduce harmful behaviors by increasing positive behaviors, ultimately shaping community values, norms, and culture.

A comprehensive, multi-level approach within the SEM is necessary to achieve population-level impact on SV (2). For over a decade, prevention scientists have emphasized that institutions should “go big or go home” by implementing comprehensive, coordinated prevention strategies (27). Offering a blended implementation strategy can address multiple levels of context and barriers of change (37). The SVPP intervenes across all levels of the SEM to address the complexity of change barriers present for students and the campus community. The curriculum integrates individual and relationship prevention approaches like social-emotional learning, consent and communication, risk-reduction, and bystander intervention skills. Other relationship and community level approaches include peer-facilitated sessions, connecting survivors to community resources and waivers, and student-led policies supporting SVPP training as a prerequisite for student organizations involvement and leadership positions. At the societal level, the SVPP aligns with state and federal laws, is grounded in public health theory, utilizes a data driven approach, and aims to challenge harmful societal norms that perpetuate and allow SV to exist within our communities.

Institutional level actions within the SEM signal the importance of prevention to the wider community. The inception of SVPP occurred in 2015 when the college's presidential initiative mandated the introduction of a comprehensive and required, four-year SV prevention curriculum for all undergraduate students. Planning and development began immediately, resulting in a launch of a multi-session first year required experience in 2019, followed by the development and implementation of a partial sophomore experience in 2020, and a complete required two-year student experience for all first years and sophomores by 2024. In terms of current program reach, for academic year 2024–2025, 96.7% (1,131/1,170) of first-year students completed all six requirements and 97.3% (1,158/1,190) of sophomores completed all four of their requirements. SVPP's annual reach was 50% (2,289/4,570) of the entire undergraduate student body. As of 2025, curriculum design and intervention piloting has begun for an upper-level SVPP experience to ultimately reach the other 50% of the student population.

While the four-year curriculum vision for the SVPP is underway, program implementation, participation, and effectiveness have been bolstered by institutional-level mechanisms for prevention. Examples of these include allocation of resources for staffing and evaluation, supportive senior leadership communications, organizational structures that focus on prevention, and conduct sanctions for non-participation. These actions support the potential impact of the SVPP.

### 2.1 The SVPP and implementation science

SVPP development, design, execution, and evaluation exists at the intersection of prevention science and implementation science. A key principle of implementation science is anchoring the development and evaluation of interventions with maximum stakeholder-engagement (27, 28). From its origin and continuing today, the SVPP has worked with students, the intended audience for the intervention, as partners and has integrated their input into the curriculum and research design. Since the creation of the Student Advisory Board (SAB), intentional recruitment strategies have aimed to reflect the diversity of the student body in SAB membership. SAB members are trained in design thinking methodology, which provides the opportunity to include their own input and collect input from peers to inform the curriculum, project and research decision making. This form of community-based participatory research ensures that a wide range of voices and perspectives help to guide the practitioners and researchers to better understand the unique needs and experiences of all students, particularly those from marginalized communities and allows us to develop more effective and relevant prevention strategies and associated evaluations (38, 39). Working with students, researchers and practitioners can better understand student motivation for program attendance and evaluation participation. For example, the SAB was instrumental in developing and testing language for curriculum activities and scenarios, guiding student communications, and identifying survey incentives and approaches to increase participation.

The inherent interdisciplinary nature of implementation science lends itself well to accessing a wide range of expertise across disciplines, which has potential to improve the quality of interventions and associated research. A particularly strong opportunity for cross-pollination that can be under-utilized in settings of higher education is that of the researchers and practitioners collaborating directly to design and implement evaluation research studies on real-world interventions. Innovative research designs for SV prevention are needed to expedite the field in moving away from ineffective interventions towards dissemination of effective offerings (27). Intentional researcher-practitioner partnerships offer an opportunity to yield such evaluation-based innovations and contribute to closing the research-practice gap (28). SVPP engagement with researchers from the field of sexual violence prevention provides a model for such innovation.

**Figure 1:**
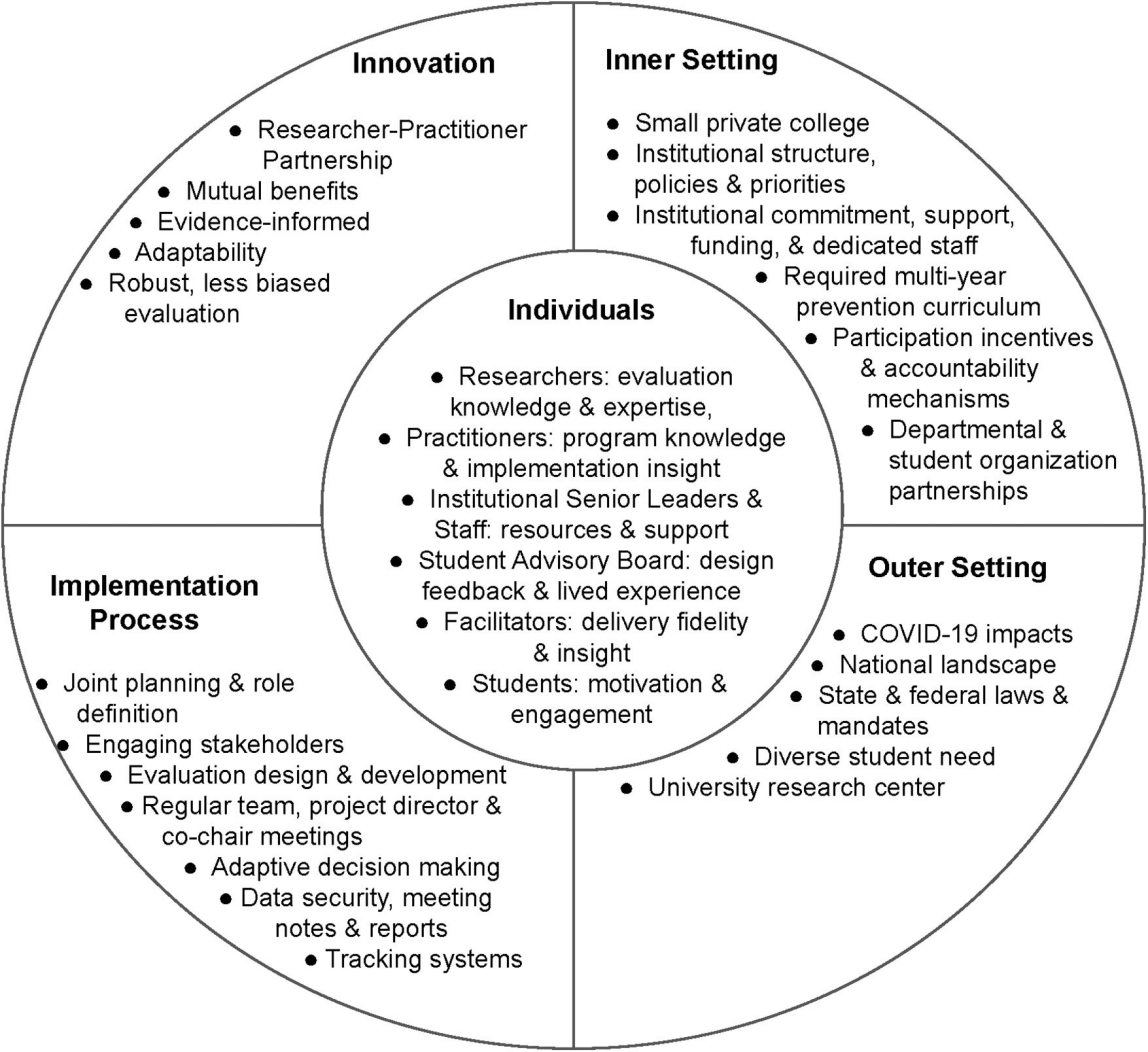
Consolidated framework for implementation research (CFIR) applied to the SVPP researcher-practitioner partnership.

By applying the CFIR to the innovation of creating a researcher-practitioner partnership to evaluate sexual violence prevention interventions, one can gain a better understanding of the complex, interacting, multi-level constructs at play. Figure 1, illuminates the richness each individual brings to the process and emphasizes the complexity of such a partnership as it relates to and is influenced by inner and outer settings.

## 3 SVPP evaluation methodology

Integrating evaluation into the design of the SVPP was a core value of the practitioner team in service of determining the overall efficacy of the project, continuous program improvement, institutionalization, and sustainability. To meet these evaluation priorities, the college contracted with a university research center and together they began planning the SVPP evaluation in the fall of 2018, early in the project's history, for a 2020 launch. Although this manuscript focuses on the partnership itself, an overview of the evaluation plan is provided for context.

The evaluation plan consisted of quality improvement non-research elements, as well as research elements. Part of the quality improvement included activities conducted by the small private college as part of their usual procedures, such as immediate post-surveys for each in-person training, debriefs with facilitators, learning artifacts collected during the sessions, and some fidelity checks. For the research activities, the researchers' Institutional Review Board (IRB) approved the research associated with the SVPP evaluation and the small private college's Committee for the Protection of Human Subjects (which serves as the IRB for the small private college) entered into a reliance agreement to accept the IRB approval of the researcher's university. The researchers focused on developing and conducting the SVPP evaluation using a mixed method design, which included adapting validated measures and creating novel instruments aligned with SVPP learning and behavioral outcomes. Meanwhile, the practitioner team managed SVPP curriculum development; facilitator training and support; program logistics, implementation and tracking; equity and inclusion considerations; fostering stakeholder engagement and campus partnerships; long-term sustainability planning; and course corrections based on the formative evaluation findings. The two teams worked with the small private college's Research, Computing and Data in the Information, Technology and Consulting office to develop, maintain, and update the secure system that allowed key de-identified information, such as consent status, to be passed to the researchers' system and integrated into longitudinal survey data collection without identifiable information.

The original research evaluation plan outlined a 5-year longitudinal outcome evaluation of students' participation in SVPP components and related changes in students' knowledge, attitudes and behaviors from pre-matriculation to post graduation. The plan also included pulse sampling of students pre and post high-risk events for instances of SV and other unwanted sexual experiences. This would have allowed for “real time” behavior assessment and additional evaluation of overall climate for undergraduates at various time points. However, several factors contributed to modifying the mixed method evaluation design. The updated evaluation plan shifted to annual formative evaluations of SVPP programmatic interventions using pre and post surveys analyzed using SPSS 27, 28, and 30; a longitudinal evaluation of students' participation in SVPP and the corresponding influence on SVPP measures through an annual survey using R; and analysis of qualitative data using thematic coding, collected through focus groups and interviews, of students' perceptions and experiences with SVPP programs. Tables 1–3 provide additional details and scope of the anticipated vs. the implemented research evaluation plans.

**Table 1:** Total survey distributions: anticipated 17, implemented 10.

Annual survey evaluation plan: anticipated vs. implemented				
Survey timepoints	Participant population	Survey distribution years (Total number of times)		Reason for adaptation N/A (not applicable)
		Anticipated	Implemented	
Timepoint 1	Incoming First Years	Pre-matriculation 2020–23 (4×)	Pre-matriculation 2020–23 (4×)	N/A
Timepoint 2	Sophomores	Fall 2020–24 (5×)	Fall 2020–24 (5×)	N/A
Timepoint 3	Juniors			N/A
Timepoint 4	Seniors			N/A
Timepoint 5	Graduating Seniors	Spring 2021–24 (4×)	Spring 2021 (1×)	Unusable response rate, logistical conflicts and barriers
Timepoint 6	Alumni 1 year after graduation	Spring/Summer 2021–24 (4×)	(0×)	Capacity, logistical conflicts and barriers

Light grey shading reflects adaptations from the original evaluation plan.

**Table 2:** Total collection points: anticipated 48, implemented 0.

Pulse sampling plan at high-risk events: anticipated vs. implemented					
High-risk events	(Total number of times) Pulse sampling years				Reason for adaptation
	Anticipated		Implemented		
1. First six weeks (first year Fall term)	Pre (4×) 2021–24	Post (4×) 2021–24	Pre (0×)	Post (0×)	COVID-19, capacity setbacks
2. Sophomore Fall Greek Recruitment	Pre (4×) 2021–24	Post (4×) 2021–24	Pre (0×)	Post (0×)	COVID-19, capacity setbacks
3. Sophomore Summer	Pre (4×) 2021–24	Post (4×) 2021–24	Pre (0×)	Post (0×)	COVID-19, capacity setbacks
4. Homecoming Weekend (Fall)	Pre (4×) 2021–24	Post (4×) 2021–24	Pre (0×)	Post (0×)	COVID-19, capacity setbacks
5. Winter Celebration (Winter)	Pre (4×) 2021–24	Post (4×) 2021–24	Pre (0×)	Post (0×)	COVID-19, capacity setbacks
6. Celebration Weekend (Spring)	Pre (4×) 2021–24	Post (4×) 2021–24	Pre (0×)	Post (0×)	COVID-19, capacity setbacks
Total collection points: 48			Total collection points: 0		

Light grey shading reflects adaptations from the original evaluation plan.

**Table 3:** Total assessments: anticipated 130, implemented 48.

Pre- and post-assessment plan for SVPP programmatic interventions: anticipated vs. implemented								
Curriculum	Distribution years (total number of times)						Programmatic Interventions x 2 (Pre & Post) x (Number of years evaluated) = Total Assessments	Reason for adaptation
First Year Experience	SAP	NSO	RS-1	PRS-1	BI-1	PEB-1		
Anticipated	2020–23 (4×)	2020–23 (4×)	2020–23 (4×)	2020–23 (4×)	2020–23 (4×)	2020–23 (4×)	6 × 2 (4×) = 48	Decided to only evaluate curriculum components tailored to SVPP curriculum outcomes; logistical barriers
Implemented	(0×)	(0×)	2020–23 (4×)	2020–23 (4×)	2021–23 (3×)	2020–22 (3×)	2 × 2 (4×) + 2 × 2 (3×) = 28	
Sophomore Year Experience			RS-2	PRS-2	BI-2	PEB-2		
Anticipated			2020–23 (4×)	2020–23 (4×)	2020–23 (4×)	2020–23 (4×)	4 × 2 (4×) = 32	COVID-19 caused setbacks in program development
Implemented			2021–23 (3×)	2021–23 (3×)	2021–24 (4×)	(0×)	2 × 2 (3×) + 1 × 2 (4×) = 20	
Junior Year Experience		SSE	RS-3	PRS-3	BI-3	PEB-3		
Anticipated		2021–23 (3×)	2021–23 (3×)	2021–23 (3×)	2021–23 (3×)	2021–23 (3×)	5 × 2 (3×) = 30	COVID-19 and staffing capacity caused setbacks in program development
Implemented		(0×)	(0×)	(0×)	(0×)	(0×)	0	
Senior Experience		SO	RS-4	PRS-4	BI-4	PEB-4		
Anticipated		2022–23 (2×)	2022–23 (2×)	2022–23 (2×)	2022–23 (2×)	2022–23 (2×)	5 × 2 (2×) = 20	COVID-19 and staffing capacity caused setbacks in program development
Implemented		(0×)	(0×)	(0×)	(0×)	(0×)	0	

SAP (Vector Solutions - Sexual Assault Prevention for Undergraduates/Athletes); NSO (New Student Orientation session); RS (Resources & Support), PRS (Positive Relationships and Sex), BI (Bystander Intervention), PEB (Power, Equity, and Belonging) with 1-4 indicating which year of the curriculum; SSE (Sophomore Summer Experience); and SO (Senior Orientation).

Light grey shading reflects adaptations from the original evaluation plan.

Several factors influenced the change in the evaluation approach. When the evaluation began, the SVPP was partially developed, with only first-year programs and one sophomore program being actively implemented. This reality necessitated a scaling back in scope since an outcome evaluation of a four-year completed comprehensive curriculum was not possible. Another substantial influential factor to adjusting the evaluation plan was the coronavirus disease 2019 (COVID-19) pandemic, which introduced a series of unanticipated disruptions that necessitated substantial revisions. One example is the unexpected need to create online versions for each in-person programmatic intervention that mirrored the original for the research. Due to the pandemic disruptions we could no longer conduct a longitudinal analysis for the programs that changed modality. Institutional shifts in priorities, resource allocation, and staffing capacity, alongside heightened health and wellness demands limited the feasibility of implementing the full anticipated evaluation design and SVPP intervention. Furthermore, the transition to remote and hybrid learning environments and restrictions on large student gatherings constrained program delivery and opportunity, reduced student participation, and hindered the collection of consistent evaluation data. These conditions challenged both the fidelity of SVPP program implementation and the reliability of established evaluation methods, requiring adaptations to measurement strategies and analysis.

The pandemic underscored the importance of flexibility in evaluation planning and highlighted the role of implementation science frameworks in guiding methodological adaptations during large-scale crises. In addition, it became clear over time that reducing the scope of the evaluation was needed, given the capacity of both teams.

The SVPP evaluation used structured collaboration between researchers and practitioners and was inclusive of stakeholder engagement, with each group sharing their expertise and learning through regular feedback loops. Early in the project, the researcher-practitioner team met with key assessment stakeholders, including the Director of Title IX and the Director of Institutional Research. The formation of an assessment team, inclusive of these stakeholders, provided comments on draft documents; proposed methods, assessment tools, and interventions; and contributed to implementation plans and data collection strategies. Additionally, the practitioners worked closely with the SVPP SAB to gather feedback on SVPP program components. The research team sought the SAB's input on program evaluation questions, recruitment strategies for engaging students to participate in the research aspects of the SVPP evaluation, and strategies to gain research consent. Finally, the researcher-practitioner team created a strong system of feedback loops by meeting weekly over the duration of the SVPP evaluation to plan alignment of evaluation research activities and program implementation, discuss formative evaluation findings, and address challenges in the execution of both the evaluation activities and SVPP program implementation. The two institutions' project directors met regularly, as did the campuses' assessment team co-chairs.

## 4 Discussion

### 4.1 Lessons learned for future applications

Practitioners and researchers both play a critical role in expanding the evidence-base in SV prevention. These partnerships can yield mutual benefits for both parties (2). Several key lessons, consistent with the literature, arose from our experience in achieving a successful researcher-practitioner partnership in college SV prevention.

### 4.2 Find and manage the right partnership

Our experience forming the SVPP evaluation partnership confirmed the literature that suggests strong, collaborative researcher-practitioner relationships need to have shared commitment, vision, expectations, and trust (30, 34). While some of the researchers and practitioners involved in this partnership had previously collaborated, the SVPP evaluation provided an opportunity for the two groups to come together to contribute their expertise, learn from other team members, and collaboratively design and execute a formative evaluation project aimed at further developing a four-year comprehensive SV prevention project. This initial information gathering process contributed to establishing shared values and a sense of operating norms. As noted by Rycroft-Malone, et al., trust is essential to productive collaborative research (34). The SVPP evaluation team worked hard to establish this trust which was strengthened by the researchers being knowledgeable about the context of SV prevention and demonstrating a strong understanding of the lived experience of practitioners, which has been shown to contribute to a more meaningful study and mutually respectful partnership (30).

As described by Nygaard and Saltz, collaborative relationships not only share a commitment to a common mission but are also characterized by a formation of new structures of both research and practice teams (40). Early in the project, the team outlined roles and responsibilities and made plans for communicating and exchanging information. Partnering with other campus entities to better understand legal, compliance, financial, and contractual obligations was essential. Establishing timelines, cadence of meetings, documentation processes, data storage, management, and security were all important elements in the creation and annual adjustment of a mutually agreed upon contract and statement of work. This early planning supported overall efficiency and ability to pivot when necessary. For instance, we discovered that key updates or decisions that only part of the team were involved with needed to be shared in a timely way with the larger group. When two people were addressing a technical issue with one of the data collection systems, others were not aware of the problem. To rectify communication gaps in a timely way, we began including the full core team on all emails, as well as keeping weekly meeting minutes that included highlights from subgroup meetings. Being able to revisit the original agreed upon goals and objectives provided clarity and an opportunity to refocus, particularly in times of challenge or change. Our process and experience confirms the proposal outlined by Rycroft-Malone, et al. that setting measurable and achievable outcomes contributes to collaboration success and facilitates monitoring, evaluation, and co-learning (34).

Finding the right partners within the research and practice communities who agree upon a shared approach and shared vision is critical.

### 4.3 Plan for adaptivity and flexibility

The reality of researching in a real-world context, especially over a long-period of time, involves facing a plethora of changes on individual, institutional, and in this case, global levels. Over the course of the partnership and research study, the SVPP evaluation team faced a multitude of decisions related to changing circumstances including staffing changes, illnesses and extended leaves, institutional leadership transitions and evolving priorities, and the COVID-19 pandemic. This variety and scale of changes in research, in which consistency is prized, can seem impossible to manage; however, with a strong researcher-practitioner relationship that has a shared understanding of the realities of implementation and a nimbleness to adapt to change, it is possible. Frameworks like the CFIR, as demonstrated in Figure 1, can help teams better understand the shifting inner and outer settings, clarify needed implementation processes, and draw upon individual characteristics to pivot effectively when needed.

Wojcik et al. describes the principle of “reflexivity” as a strategy for building successful partnerships that encourages researchers to adjust methods based on input from practitioners (36). The SVPP evaluation team practiced reflexivity throughout the study that resulted in restructuring the evaluation plan to be more condensed and realistic, refining assessment questions based on updated programming, improving data collection methods, and contributing to a more well-rounded analysis.

To achieve this, a version of a Community of Practice (CoP) was formed in which members from different aspects of the project shared viewpoints, discussed common issues, explored ideas, and offered solutions to challenges. CoPs are a popular mechanism for helping disseminate evidence-based practices through collaboration by bringing together individuals from different professions to share perspectives, problem-solve, and deepen expertise (35). The SVPP evaluation CoP allowed for rapid cycle integration of feedback and changes by embracing processes such as the Plan, Do, Study, Act (PDSA) model for quality improvement (41, 42). For instance, after the conclusion of each post-survey collection point, all parties would bring data and interpretations to a collaborative meeting. The practitioner team would provide immediate post-survey feedback regarding student participants' experience of the facilitator and the workshop, synthesized observations from the facilitators, and any related input from the SAB. The researcher team would provide pre-post survey statistical analysis and other data collected through the research instruments. Collaborative conversations anchored in openness, curiosity, and shared goals allowed for better clarification and interpretation of all available data which in turn informed decision making, such as adapting recruitment strategies for program post surveys.

With a researcher-practitioner partnership, in which both parties understand and expect real-life research conditions that demand a certain amount of flexibility, systems can be created to capitalize on the collective expertise to inform decision-making. While the process may be less consistent than controlled-settings, this type of practice-to-research project is more reflective of implementation realities, therefore making results more applicable and meaningful.

Both parties, when researching in real-world context, must bring flexible mindsets and be willing to adapt program and evaluation elements as needed.

### 4.4 Co-create systems and processes for navigating complex logistics

The SVPP evaluation team created and leveraged a system to track both the programmatic and evaluation activities, as one example of implementation processes found in Figure 1. These systems allowed the team to monitor program implementation and collection of evaluation data in real time. This tracking system was managed by one member from both the practitioner and researcher partner institutions respectively. The tracking spreadsheet was updated regularly and reviewed during most team meetings. This documentation allowed the SVPP evaluation team to make informed decisions on the timing of program implementation, recruitment for pre and post program surveys, and submission of IRB materials. It also provided deadlines for the completion of SVPP program development, adjustments to survey recruitment strategies, and modifications to survey instruments.

Because prevention research is often done in a controlled or experimental setting, collaborations with practitioners can increase researchers' understanding of appropriate evaluation plan designs for real world settings as well as better alignment of corresponding tools and measures with prevention programs being implemented (40). Practitioners can become important “informants” for researchers helping to guide what is and isn't working with the evaluation strategies. Researchers through the evaluation process can inform practitioners of what is and isn't effective in their programming. This feedback loop can ultimately increase resources and sustainability towards prevention efforts that yield meaningful results.

For instance, partially through the evaluation, response rates for post-survey data collection were extremely low, which resulted in creating a new data collection process. After consulting the SAB, we learned that students placed low value on the raffle and low-level incentives being used. Therefore, we replaced the incentive-driven approach with a convenience-driven approach. We began administering post surveys at the beginning of the next training when students were present and already giving their time towards the project. The orchestration of this change was managed by both teams. The practitioners adjusted the timing of the programs by five minutes, communicated the change in the post survey implementation and incentives, and added transition scripts for program facilitators. The researchers managed the release of the links to the online post surveys, monitored response trends, and updated the team weekly on increases in response rates. The changes led to increases in post-survey participation. For example, we saw a 209% increase in matched pairs response rates (from *N* = 120 to *N* = 371) in our Positive Relationships and Sex program for first years from academic year 2022–23 to academic year 2023–24 after the procedural changes took place. With strong communication and willingness from both research and practice teams, the adjustments, informed by students, yielded an increase in response rates while also saving financial resources.

Clear mechanisms and processes contribute to the ability to build trust and communicate with respect across roles.

### 4.5 Build trust by honoring expertise and clarified roles

The SVPP evaluation team needed to balance evaluation expertise with prevention expertise and vice versa. The research team had a strong background in evaluation and SV curriculum development. As such, they were careful to provide evaluation findings within the context of high-level programmatic recommendations so that they would not influence or interfere with the practitioners' program development. For example, during the first year of the project, program evaluations indicated that students were gaining knowledge from a certain program, but not the skills that aligned with the program's learning objectives. Based on these findings, the researchers recommended that the practitioners increase skill development in the program and realign the curriculum with the learning objectives. They did not suggest specific skill-building activities or how to align curriculum content with learning objectives. Instead, based on the evaluation findings, the practitioner team worked together to revise the curriculum elements to further support skill development.

Similarly, the practitioner team had a staff member dedicated to evaluation and other team members had participated in research and evaluation projects. This evaluation expertise from the practitioner team strengthened the overall evaluation and contributed to informed conversations; however, the practitioners were careful not to influence the outcomes of the evaluation by projecting their intentions for the programs into the evaluation questions, design, and findings. Leaning on the strengths and expertise of the research team as independent evaluators was key in deferring final evaluation decisions to them.

The team also navigated when to make changes to the SVPP programs. After shifting to a formative approach to the evaluation, we could intentionally use the evaluation findings for program improvement. We still had to balance practitioners' desire to alter program content based on students' feedback with the consistency needed in research. Thus, program content was not changed until all of the programs had been delivered for that year. This helped ensure fidelity to the programs' curriculum elements and learning objectives. When changes to the intervention did occur the following year, we sacrificed the ability to have comparable data across years. Through feedback loops described above, we were able to leverage the formative evaluation for continuous program improvement while staying true to the evaluation design. This required trust, both in the evaluation process and in the researcher-practitioner partnership. This trust was built, maintained and sustained by consistent communication, humility and commitment to carrying out the goals of the SVPP evaluation.

These examples demonstrate the SVPP evaluation team members' ability to bring all their areas of expertise to the collaboration while at the same time acknowledge their individual roles by “staying in their lane” to ensure integrity in the evaluation process and fidelity to program implementation. The team valued each other's expertise and roles and worked to support one another. This letting go of control and trusting the evaluation process helped both individual teams invest in a data-driven and real-world implementation of the SVPP evaluation. The collaboration was made possible because the individuals and the two teams engaged in open, respectful and consistent communication. The success of this collaboration, according to Sullivan et al, is grounded in the team having a shared vision, jointly determined expectations that are rooted in the skills of the partners, and open communication (30). Honoring the expertise of both parties and aligning activities accordingly can contribute to a more objective and balanced assessment of program effectiveness. This less-biased analysis can give credibility to findings, potentially leading to attention, resources, and appreciation for staff-developed effective programming.

Research and practitioner partners must be willing to share the work, contributing equally and with humility with an emphasis on each team's specified strengths and roles.

## 5 Conceptual and methodological constraints

When we began the evaluation, we had an elaborate longitudinal evaluation plan using multiple evaluation methods. The pandemic occurred in our first year and required us to completely reshape our evaluation plan from focused on longitudinal outcomes towards a more formative design and also shifting our methods. We had to eliminate pulse sampling and minimize interviews and focus groups, predominantly relying on quantitative surveys with qualitative questions as our primary methods of data collection. In part, this was also due to the partnership creating an original plan that was ideal, but in reality, unrealistic, given staffing levels on both sides.

Some of the prevention programs in the SVPP curriculum were not fully developed at the time of the evaluation, which presented challenges to finalizing the full evaluation plan, creating consistent measures, and writing IRB documents under required timelines.

The SVPP program attendance tracking system was also being built as the evaluation began and changed platforms during the evaluation. This created challenges of tracking students' data over time, including ensuring all historical data was transferred and linked properly.

We acknowledge the limitations of the generalizability of our experience as a single private college partnering with external research experts.

## 6 Conclusion

To bridge the research to practice gap, researchers and practitioners must work collaboratively on evaluation projects not just as a technical asset but a relational one grounded in shared goals, clear roles and expectations, and mutual respect. The SVPP evaluation was created by researchers and practitioners partnering together to design and implement an evaluation approach robust enough to inform programmatic decisions, while also accommodating shifting contexts, including a pandemic that necessitated rapid changes in the evaluation plan, delivery formats, dosages, and engagement strategies. Across the partnership, we employed implementation science principles to navigate the turbulence of real-world challenges. Our joint openness to designing and delivering the evaluation plan considering context and with strong stakeholder engagement, along with creating feedback loops founded in open communication and trust contributed to our ability to assess evidence-based strategies at the core of the SVPP and supported continuous quality improvement of the program.

Combining practitioner insight and experience with expert research design and knowledge increases the ability to understand and expand practices that can have a measurable impact on student wellbeing and decrease violence. Researcher-practitioner collaborations exist as an available resource that can effectively measure promising prevention strategies anchored in the realities of campus life. Institutions supporting researcher-practitioner collaborations demonstrate a dedication to addressing SV by actively engaging in evidence-based evaluation, ensuring long-term commitment to effective prevention.

## Data Availability

The original contributions presented in the study are included in the article/Supplementary Material, further inquiries can be directed to the corresponding author.
